# Weight Gain in Seropositive Rheumatoid Arthritis Patients Treated With Anti-tumor Necrosis Factor (TNF) Therapy

**DOI:** 10.7759/cureus.25556

**Published:** 2022-06-01

**Authors:** Toka M Alsulaim, Maryah H Almulla, Najla S Alotaibi, Elham A AlQudari, Khalid A Alzahrani, Rawad M Nori, Mosaab a Makkawy, Hanan A Alhamzi

**Affiliations:** 1 Rheumatology, Prince Mohammad bin Abdulaziz Hospital, Riyadh, SAU; 2 Rheumatology, Prince Mohammad bin Abdulaziz Hospital, Riyadh, SAU; 3 Biostatistics, King Fahad Medical Research Centre, Jeddah, SAU

**Keywords:** non-tnf, inhibitors, anti tnf, body weight, rheumatoid arthritis

## Abstract

Introduction

Rheumatoid arthritis and its treatment have different effects on weight gain. This study examined the effect of tumor necrosis factor (TNF) inhibitors on weight gain in patients with seropositive rheumatoid arthritis compared with non-TNF therapy and disease activity.

Methods

A retrospective cohort study was conducted in Prince Mohammad bin Abdulaziz Hospital for all patients aged ≥ 18-year-old diagnosed with seropositive rheumatoid arthritis and started on TNF inhibitors or non-TNF biologics in outpatient clinics since 2015. Patients were excluded if they were pregnant, had an uncontrolled underlying metabolic disease, were post-bariatric, or had a history of malabsorption. We also excluded individuals on treatment for congestive heart failure or end-stage renal disease.

Results

A total of 116 patients with rheumatoid arthritis were reviewed between 2015 and 2019. Only 69 patients met the inclusion criteria (51 and 18) in the TNF-a (alpha) and non-TNF-a inhibitor groups, respectively. The weight change from pre-treatment to post-treatment showed an average increase of 1.2 kg (95% CI: -0.68-3.17) in the TNF-a inhibitor group while in the non- TNF-a inhibitor group, the average increase in weight was 2.67 kg (95% CI: -0.44-5.78). Over the period of two years, there was no statistical difference in weight gain in both groups or in relation to disease activity.

Conclusion

The results of this study did not show a significant increase in weight gain in seropositive patients with rheumatoid arthritis who were treated with TNF or non-TNF inhibitors.

## Introduction

Tumor necrosis factor-alpha (TNF-a) is expressed in each cell in our body. It plays a significant role in the pathophysiology and disease activity of various inflammatory diseases such as but not limited to rheumatoid arthritis (RA) and psoriatic arthritis, along with inflammatory bowel disease and ankylosing spondylitis. That explains its different activities and role in regulating the human body interaction. TNF-a inhibitors are used in patients with RA who failed to reach disease control by non-biological therapies [1].

Overweight and obese were estimated to be around 35% of the world population. It was linked that this increasing prevalence was associated with a higher risk of negative impact on quality of life [2].

In patients with rheumatoid arthritis, a higher body mass index (BMI) has been linked to worse outcomes in both disease activity and function [3].

The effect of anti-TNF on weight is not well-established and is yet to be determined in correlation to disease activity. Different studies have shown the impact of TNF inhibitors on weight gain with variable and heterogeneous data; however, till now, we have no clear answer for the effect of these medications on seropositive patients with rheumatoid arthritis. We have also considered that seronegative rheumatoid arthritis can have some diagnostic confusion [1-4].

This is the first study in the Middle East and North Africa where the effect of TNF inhibitors on weight gain in a specific group of patients (only seropositive rheumatoid arthritis) has been studied, and this result was compared and correlated with non-TNF therapy and disease activity.

## Materials and methods

We conducted a retrospective cohort study using data from Prince Mohammad bin Abdulaziz Hospital Integrated Clinical Information System by collecting data of adult rheumatology patients diagnosed with seropositive rheumatoid arthritis and following in outpatient clinics since 2015. 

The study was conducted in accordance with the ethical principles of the Helsinki Declaration and Prince Mohammad bin Abdulaziz Hospital, Ministry of Health, policies and guidelines for clinical research. The patient's identity was protected and known only by the investigators. 

We identified all adult patients aged ≥ 18-year-old with a confirmed diagnosis of seropositive (positive serum cyclic citrullinated peptide or rheumatoid factor or both) rheumatoid arthritis who will be treated with TNF-a inhibitors and will be compared to those who receive non-TNF-a inhibitor therapy (non-TNF biologics and small-molecule inhibitors) as a control group at Prince Mohammad bin Abdulaziz Hospital Rheumatology Clinics.

Patients' weight, along with their other demographics, the time of treatment initiation, disease activity assessment by Disease Activity Score (DAS-28), and type of treatment, were collected from the system every three to six months for 24 months from TNF-and non-TNF initiation.

Patients were excluded if they were pregnant, had an uncontrolled underlying metabolic disease, were post-bariatric, or had a history of malabsorption. We also excluded individuals on treatment with congestive heart failure or end-stage renal disease (ESRD).

The primary outcome was the change in body weight in patients initiated on TNF-a inhibitors compared with non-TNF-a inhibitors treated patients over the study period. 

Secondary outcomes were to assess the difference between TNF-a and non-TNF-a inhibitor groups regarding body weight change and to assess the relationship between the initiation of TNF-a inhibitors, weight change, and disease activity. 

Statistical analysis

Descriptive statistical parameters were reported as mean ± standard deviation (SD) for continuous variables and n (%) for categorical variables. To assess the normal distribution of data, The Shapiro-Wilk normality test was used. The study groups at baseline were compared using T-tests for continuous variables and chi-square (χ2) analysis for categorical variables. The T-test was used to compare pre and post-treatment weight changes in both groups. P < 0.05 was considered significant. All statistical analyses were performed using RStudio software (version 3.3.0; RStudio Team (2020). RStudio: Integrated Development for R. RStudio, PBC, Boston, MA. http://www.rstudio.com/).

## Results

A total of 116 patients with RA were reviewed between 2015 and 2019. Only 69 patients met the inclusion criteria (51 and 18) in the TNF-a inhibitor and non-TNF-a inhibitor groups, respectively. Baseline characteristics were identical between the two groups (Table 1).

**Table 1:** Baseline characteristics Abbreviations: cDMARDS: classical disease-modifying anti-rheumatic drugs, HTN: hypertension, DM: diabetes mellitus, SD: standard deviation MTX: methotrexate. LEF: leflunomide Values are mean and proportion % (in parentheses) unless specified otherwise. ^a^ P-values were based on the chi-square test for categorical variables the and t-test for continuous variables

Characteristics n (%)	Anti-TNF-α (n=51)	Non-anti-TNF-α (n= 18)	P-value ^a^
Age, in years, mean ± SD	50.2 ± 12.6	54.7 ± 12.0	.177
Male	7 (13.7)	4 (20)	.456
Female	44 (86.3)	14 (80)	
MTX	18 (35.3)	3(17)	
LEF	1 (1.9)	0	
Steroids: No	41 (80.4)	9 (50)	.145
Steroids: Yes	10 (19.6)	9 (50)	
Anti-HTN	3 (5.9)	1 (5.6)	
Anti-DM	5 (9.8)	0	
Lipid-lowering agents	4 (7.8)	1 (5.6)	
Hormonal therapy	3 (5.9)	0	
No other medications	29 (56.9)	7 (38.9)	

Female patients formed most of the enrolled, 86.3% in the TNF-a inhibitor group and 80% in the non-TNF-a inhibitor group. Twenty-nine (29; 56.9%) patients in the TNF-a inhibitor group were not on any active medications for other comorbidities compared to only seven (38.9%) from the non-TNF-a inhibitor group.

On the other hand, comorbidities commonly reported in both groups were hypertension, diabetes, thyroid disease, and chronic kidney disease in 26.1%, 21.7%, 5.8%, and 4.3% of patients, respectively. There was no effect of baseline prednisolone, anti-hypertension, diabetes medications, and thyroid hormone on weight gain.

We reported 19 (37.3%) patients on adalimumab, 30 (58.8%) patients on etanercept, and two (3.9%) patients on infliximab. In the non-TNF-a inhibitor group, data showed three (20%) patients on abatacept, four (20%) patients on rituximab, five (25%) patients on tocilizumab, and six (35%) patients on tofacitinib treatment.

The body weight documented at baseline and then at follow-up at different time intervals over 24 months was collected (Table 2).

**Table 2:** Bodyweight baseline follow-up and disease activity score stratified by treatment groups

Parameters	Anti-TNF-α mean ± SD	Non-anti-TNF-α mean ± SD
Weight in kg		
MONTH 0	74.4 ± 16.9	73.2 ± 16.9
MONTH 3	74.9 ± 17.4	73.8 ± 16.9
MONTH 6	76.2 ± 15.4	74.9 ± 16.3
MONTH 12	76.9 ± 15.5	77.8 ± 19.4
MONTH 18	75.8 ± 16.4	77.9 ± 18.3
MONTH 24	75.2 ± 15.4	77.1 ± 16.2
Disease activity score		
MONTH 0	4.2 ± 1.2	4.9 ± 1.2
MONTH 3	2.6 ± 1.1	3.3 ± 0.7
MONTH 6	2.7 ± 1.3	3.3 ± 1.2
MONTH 12	2.5 ± 1.1	3.5 ± 1.5
MONTH 18	2.6 ± 1.1	2.7 ± 1.1
MONTH 24	2.7 ± 1.1	2.5 ± 1.0

The disease activity collected at the same intervals demonstrated as mean ± standard deviation (Table 3).

**Table 3:** Disease active/remission status and weight change over time ^a^ P-values were based on a two-sample t-test.

Duration	Anti-TNF-α			Non-anti-TNF-α		
	Active	Remission	P-value ^a^	Active	Remission	P-value ^a^
MONTH 3	69.4 ± 19.6	76.9 ± 16.7	0.309	73.7 ± 22.8	73.9 ± 15.6	.991
MONTH 6	78.9 ± 13.9	74.5 ± 16.4	0.416	87.0 ± 17.1	70.2 ± 13.9	.144
MONTH 12	77.7 ± 14.2	76.3 ± 15.9	0.823	78.9 ± 10.7	76.3 ± 28.5	.834
MONTH 18	72.1 ± 13.9	77.8 ± 17.5	0.231	81.7 ± 14.2	75.6 ± 21.6	.649
MONTH 24	76.6 ± 13.8	74.8 ± 16.1	0.742	85.4 ± 23.0	73.6 ± 12.9	.473

The mean weight at enrollment was 74.4 kg and 73.2 kg; it increased to 75.2 kg and 77.1 kg at the end of the study period for the TNF-a inhibitor group and non-TNF-a inhibitor group, respectively. The weight change from pre-treatment to post-treatment showed an average increase of 1.2 kg (95% CI: -0.68-3.17) in the TNF-a inhibitor group while in the non-TNF-a inhibitor group, the average increase in weight was 2.67 kg (95% CI: -0.44-5.78). The conducted paired-sample t-test showed no significant difference in the mean weight pre and post-treatment in the TNF-a inhibitor group (P=0.199) and non-anti TNF groups (P=0.084).

Over the period of two years, there was no statistical difference in weight gain between the two groups or in the same group. However, numerically, there was more weight gain in the non-TNF group compared to the TNF group.

There was no statistically significant difference between TNF and non-TNF regarding disease activity. There was no significant statistical relationship between disease activity and weight gain, though there was a numerically proportional relationship between weight gain and improving disease activity (Tables 2-3).

## Discussion

Rheumatoid arthritis can affect around 1% of the population in the world. Chronic synovial inflammation can lead to joint destruction and function loss [5].

The body weight in patients with rheumatoid arthritis independently impacts the disease activity, response to treatment, and quality of life [2,6]. Our pooled data noted that there was no significant correlation between weight and response to treatment therapy. Two-thirds of rheumatoid arthritis patients are overweight or have obesity. Weight gain in such a group of patients can be associated with higher disease activity and poor response to treatment [7].

TNF-a inhibitors are one of the biological therapies that we used in RA patients to control the disease if non-biological therapy did not work [8]. They were manufactured to suppress TNF-a's pro-inflammatory properties. They are divided into either monoclonal antibodies that include adalimumab, golimumab, infliximab, certolizumab pegol, or receptor fusion protein (etanercept). When approved for treatment, the alternation of TNF-a function was associated with adverse effects ranging from infection, malignancy, autoimmune disorders, and, to a lesser extent, metabolic disturbances and weight changes [6,9].

In a recent analysis in 2020 by Patsalos O et al., 26 studies had been collected to look for the effect of anti-TNF on weight and BMI. There was a small increase in weight and BMI with diverse data (0.90 kg, 2.34 kg, and 2.27 kg) with infliximab, etanercept, and adalimumab, respectively. However, they included different disease groups (rheumatoid arthritis, inflammatory bowel disease, psoriasis, and ankylosing spondylitis) that can affect body weight by different mechanisms [9].

A meta-analysis by Wu et al. on psoriatic patients showed a significant increase in body weight on anti-TNF initiation in comparison with interleukin (IL) 12/23 and IL-17 [10].

Serelis et al. studied the effects of anti-TNF-α treatment in 19 women with rheumatoid arthritis, showing no significant impact on body composition; however, there was an increase in adiponectin levels that can affect the systemic inflammation [11]. Conversely, in a randomized study during a follow-up of 21 months, Engvall et al. showed that in 40 patients with RA, the body fat mass increased significantly on infliximab therapy [12].

The mechanism by which TNF-a inhibitors affect weight is not well-understood, but TNF may inhibit lipoprotein lipase, leading to weight loss in RA patients [13].

In RA patients who gain weight with TNF inhibitor (infliximab and etanercept) treatment, leptin and adiponectin levels have elevated [12,14]. In RA patients who were treated with tocilizumab, adiponectin level was increased with increased lean mass [4].

In contrast to previous research with heterogeneous patient populations, we eliminated seronegative rheumatoid arthritis to eliminate this heterogeneity. The effect of anti-TNF medication versus non-TNF therapy (biologics and small targeted compounds) on weight gain in patients with rheumatoid arthritis are compared. In our study, the data follow-up was for two years.

In this retrospective study, there was a non-significant statistically increase in body weight over time. However, numerically, there was an increase in weight gain over therapy with non-TNF medications and to a lesser extent with the TNF group. That increase in body weight may also reflect an improvement in disease activity although it was not statistically significant.

Till now, the exact mechanism of weight gain during rheumatoid arthritis treatment has not been well-studied. By reviewing the previous studies and with our study findings, we postulated the following hypothesis: In patients who gain weight, inhibition of inflammatory cytokines may enhance adipose tissue proliferation, which increases both adiponectin and leptin production. With time, this can cause a lack of sensitivity to both, leading to leptin and adiponectin resistance. Because the individual keeps eating, the fat cells produce more leptin to signal the feeling of satiety, leading to increased levels of both hormones. Another hypothesis is that neutralizing antibodies against both leptin and adiponectin can develop during biological therapy by looking at some studies showing an increase in adiponectin levels without an increase in body weight [11].

## Conclusions

The results of this study did not show a significant increase in the weight gain in seropositive rheumatoid arthritis patients who were treated with TNF or non-TNF inhibitors. However, numerically, there was a trend of weight gain in both groups, which was more in the non-TNF groups. This increase was going with improvement in disease activity. The effect of rheumatoid arthritis and treatment on weight gain is variable and needs to be monitored during treatment.
